# Downregulation of α7 nicotinic acetylcholine receptors in peripheral blood monocytes is associated with enhanced inflammation in preeclampsia

**DOI:** 10.1186/s12884-019-2340-5

**Published:** 2019-05-28

**Authors:** Hongmei Xu, Qingyang Shi, Yanxiang Mo, Linlin Wu, Jishuang Gu, Ying Xu

**Affiliations:** 1 Department of Obstetrics, the First Hospital of Jilin University, Changchun, Jilin China; 2 Center for Prenatal Diagnosis, the First Hospital of Jilin University, Changchun, Jilin China; 3 Department of Nephrology, the First Hospital of Jilin University, Changchun, Jilin China

**Keywords:** Preeclampsia, α7nAChR, Hypertension, Inflammation, NF-κB

## Abstract

**Background:**

Preeclampsia is associated with chronic inflammation. The cholinergic anti-inflammatory pathway regulates systemic inflammation through activating α7 nicotinic acetylcholine receptors (α7nAChR) expressed in monocytes/macrophages. This study aimed to investigate the role of α7nAChR in peripheral blood monocytes in preeclampsia.

**Methods:**

Peripheral blood monocytes were isolated from 30 nonpregnant (NP), 32 normotensive pregnant (NT), and 35 preeclamptic (PE) women.

**Results:**

We found that both protein and mRNA expression levels of α7nAChR in monocytes from the PE women were significantly lower than those of the NP and NT women (both *p* < 0.01). α7nAChR protein expression levels in monocytes were negatively correlated with levels of systolic blood pressure (r = − 0.40, *p* = 0.04), proteinuria (r = − 0.54, *p* < 0.01), tumor necrosis factor-alpha (TNF-α, r = − 0.42, *p* = 0.01), and interleukin (IL)-1β (r = − 0.56, *p* < 0.01), while positively correlated with IL-10 levels (r = 0.43, *p* = 0.01) in the PE women. Both baseline and lipopolysaccharides (LPS)-induced increase of TNF-α, IL-1β, and IL-6 levels from monocytes were higher in the PE group than the NP and NT groups (all *p* < 0.01), but IL-10 levels in the PE group was lower than that of the NP and NT groups (*p* < 0.01). In addition, the NF-κB activity in monocytes from the PE women was higher than the NP and NT women (*p* < 0.01). Importantly, activation of α7nAChR with its agonist PNU-282987 inhibited NF-κB, decreased TNF-α, IL-1β, and IL-6 release, and increased IL-10 release in monocytes from the PE women (all *p* < 0.01).

**Conclusion:**

In conclusion, these findings suggest that downregulation of α7nAChR may be associated with the development of preeclampsia through increasing pro-inflammatory and decreasing anti-inflammatory cytokine release via the NF-κB pathway.

## Introduction

Preeclampsia is a common and serious condition during pregnancy, which is characterized by pregnancy-induced hypertension and proteinuria after 20 weeks of pregnancy. Approximately 5% of all pregnancies worldwide are affected by preeclampsia. Preeclampsia is a major cause of pregnancy-related morbidity and mortality [1].

Preeclampsia is associated with chronic systemic inflammation with activation of immune cells and increased inflammatory cytokines [2]. It has been reported that peripheral blood monocytes are highly activated in pregnant women with preeclampsia [3–5]. For instance, increased pro-inflammatory subsets of peripheral blood monocytes are correlated with the severity of preeclampsia [6]. Furthermore, peripheral blood monocytes from preeclamptic women are preferably polarized to a M1 pro-inflammatory macrophage phenotype with increased tumor necrosis factor-alpha (TNF-α) and decreased interleukin (IL)-10 levels [7]. Preeclampsia is also associated with increased macrophage infiltration in the placenta with increased release of pro-inflammatory cytokines [8, 9]. It is speculated that activation of peripheral blood monocytes contributes to enhanced inflammatory responses in placental tissue of preeclamptic women. However, the mechanism underlying the activation of peripheral blood monocytes in preeclampsia is largely unknown.

The cholinergic anti-inflammatory pathway regulates systemic inflammatory responses and plays an important role in a variety of inflammation-related disorders, such as hypertension and kidney diseases [10–12]. In the pathway, acetylcholine activates α7 nicotinic acetylcholine receptors (α7nAChR) expressed in monocytes/macrophages [13–15]. Activation of α7nAChR regulates monocyte and macrophage polarization and suppresses expression of pro-inflammatory cytokines, including TNF-α and IL-1β, probably through inhibition of nuclear factor-κB (NF-κB) transcription activity [13, 16]. It was reported that α7nAChR downregulation is involved in activation of peripheral blood monocytes in various pathological conditions [17–19]. However, it is unknown whether α7nAChR is involved in activation of peripheral blood monocyte in pregnant women with preeclampsia.

There is evidence showing an association between α7nAChR and preeclampsia. In pregnant rats, activation of α7nAChR by nicotine mitigated lipopolysaccharide (LPS)-induced placental inflammation and symptoms mimicking preeclampsia likely through suppressing the NF-κB p65 pathway [20]. Previous studies demonstrated that expression of α7nAChR in the placenta was increased in pregnant women with preeclampsia compared with nonpregnant women [21–23]. These findings suggest that expression of α7nAChR may be associated with placental inflammation and the development of preeclampsia. However, the role of α7nAChR in the activation of peripheral blood monocytes in preeclampsia is unclear.

Taken together, we hypothesized that the α7nAChR expression levels are decreased in peripheral blood monocytes of preeclamptic women and that the downregulation of α7nAChR is associated with monocyte activation, enhanced inflammation, and the severity of preeclampsia. In this study, we tested the hypothesis by conducting ex vivo and in vitro experiments using isolated peripheral blood monocytes from pregnant women with preeclampsia, normotensive pregnant women, and nonpregnant women.

## Methods

### Study population

The Institutional Review Board of The First Hospital of Jilin University reviewed and approved the study protocol (16Y158–002). Written informed consent was obtained from all participants. The study consists of 32 normotensive and 35 preeclamptic pregnant women in the third trimester of their pregnancy and 30 age-matched nonpregnant women. Preeclampsia was defined as a combination of hypertension (≥140/90 mmHg evaluated on two consecutive occasions) after the 20 weeks of gestation and proteinuria of ≥300 mg in urine collected during 24 h [24, 25]. Severe preeclampsia was diagnosed by the presence of one or more of the following: systolic blood pressure ≥ 160 mmHg or diastolic blood pressure ≥ 110 mmHg; thrombocytopenia; impaired liver function; progressive renal insufficiency; pulmonary edema; new-onset cerebral or visual disturbances [25]. Among the 35 preeclamptic pregnant women, 20 had mild preeclampsia and the other 15 had severe preeclampsia.

### Sample collection and preparation

Before any medications were given, peripheral venous blood was drawn from the subjects by venipuncture and collected into heparinized tubes at the time of disease diagnosis for the normotensive and preeclamptic pregnant women and during a health checkup visit for nonpregnant women. CD14^+^ monocytes were isolated immediately from the fresh whole blood samples with Ficoll’s density gradient centrifugation and subsequent magnetic-activated cell sorting as described previously [26].

### Flow cytometry

To study levels of surface α7nAChR protein expression, the isolated monocytes were stained with anti-α7nAChR antibody (1:100, ab216485, Abcam, Cambridge, MA, USA) for 30 min at 4 °C. After washed with buffer containing 1% bovine serum albumin, the cells were incubated with Alexa 488-conjugated anti-rabbit IgG (1:500, ab150077, Abcam, Cambridge, MA, USA) for 15 min at room temperature. The samples were analyzed on a flow cytometer (BD Biosciences). Mean fluorescence intensity was used to quantify the surface α7nAChR protein expression level [27].

### Real-time polymerase chain reaction (RT-PCR)

Total RNA was extracted from the isolated monocytes with TRIzol reagent. RT-PCR was performed using One Step SYBR Prime Scrip RT-PCR Kit II (TaKaRa). PCR primer sequences are listed as follow: α7nAChR: F, 5′-ACA TGC GCT GCT CGC CGG GA-3′; R, 5′-GAT TGT AGT TCT TGA CCA GCT-3′; and GAPDH: F, 5′-GTC GCT GTT GAA GTC AGA GG-3′; R, 5′-GAA ACT GTG GCG TGA TGG-3′. The α7nAChR mRNA expression was calculated by 2^−ΔΔCT^ and normalized to GAPDH. The levels of α7nAChR mRNA expression in nonpregnant women were set to a value of 1. Each sample was run and analyzed in triplicate.

### Monocyte culture

The isolated monocytes were cultured in 24-well flat-bottomed plates with RPMI 1640 medium at the density of 1 × 10^6^ cells/mL. After incubated with complete medium for 18 h, supernatant was collected for measurement of cytokines. To study stimulated cytokine release, monocytes were treated with LPS (1 ng/mL, Sigma-Aldrich, St Louis, MO, USA), and supernatant was collected for measurements of cytokines at 12, 24, and 48 h after LPS treatment. In another set of experiments, monocytes were treated with or without an α7nAChR agonist PNU-282987 (PNU, 10 μmol/L, Sigma-Aldrich, St Louis, MO, USA) for 24 h; then supernatant was collected for cytokine and cells were harvested for NF-κB activity measurements. PNU is a highly selective α7nAChR agonist (Ki = 26 nM). In addition, monocytes were treated with or without an NF-κB inhibitor BAY 11–7085 (10 μmol/L, Sigma-Aldrich, St Louis, MO, USA) for 24 h, and then cells were collected for NF-κB activity measurements. BAY 11–7085 prevents activation of NF-κB through irreversibly inhibiting IκBα (the inhibitor of NF-κB) phosphorylation (IC50 = 10 μM).

### Enzyme-linked immunosorbent assays (ELISA)

Protein expression of TNF-α, IL-1β, IL-6, and IL-10 in the culture medium of monocytes collected from above experiments was measured by ELISA kits (ab181421, ab46052, ab46027, ab46034, Abcam, Cambridge, MA, USA).

### NF-κB transcription activity assay

Nuclear fractions of treated monocytes were extracted, and the DNA binding activity of NF-κB was detected using NF-κB p65 Transcription Factor Assay Kit (ab133112, Abcam, Cambridge, MA, USA) in accordance with manufacturer’s instructions.

### Statistical analysis

Statistical analysis was performed with GraphPad Prism 6.0 software (GraphPad Software Inc. San Diego, CA, USA). Continuous data are presented as mean ± standard deviation and analyzed by one-way analysis of variance (ANOVA) or two-way ANOVA followed by Bonferroni post-test if the data had a normal distribution. Categoric variables were expressed as number and percentage of patients and analyzed with Chi-square test. Pearson correlation and linear regression were performed to examine the relationship between two parameters. Differences were considered significant at a probability level of *p* < 0.05.

## Results

### Patient characteristics

The demographic and clinical characteristics of the subjects are presented in Table 1. Age was comparable among nonpregnant, normotensive pregnant, and preeclamptic pregnant women, and gestational age and parity were similar between normotensive and preeclamptic pregnant women (Table 1). Systolic and diastolic blood pressure levels were similar between nonpregnant and normotensive pregnant women but were significantly lower in these two groups than pregnant women with preeclampsia (Table 1). Levels of proteinuria and uric acid of preeclamptic women were 2.6 ± 0.5 g/24-h and 6.2 ± 0.6 mg/dL, respectively, whereas proteinuria and uric acid levels of nonpregnant and normotensive pregnant were within normal ranges (Table 1).

**Table 1:** Characteristics of nonpregnant, normotensive pregnant, and preeclamptic women

Variable	Nonpregnant (*n* = 30)	Normotensive (*n* = 32)	Preeclamptic (*n* = 35)
Age, years	27.3 ± 4.3	26.3 ± 2.8	26.7 ± 5.1
Gestational age (weeks)	N/A	35.5 ± 1.6	35.1 ± 1.5
Parity	N/A	0.22 ± 0.42	0.20 ± 0.41
Systolic BP (mmHg)	107.6 ± 5.3	108.8 ± 7.1	157.1 ± 7.6**
Diastolic BP (mmHg)	67.5 ± 4.5	68.2 ± 5.5	107.6 ± 7.1**
Proteinuria (g/24 h)	< 0.3	< 0.3	2.6 ± 0.5
Uric acid (mg/dL)	3.6 ± 0.6	3.9 ± 0.7	6.2 ± 0.6**

Data are mean ± standard deviation. *BP* blood pressure. Data were analyzed using one-way ANOVA followed by Bonferroni post-test. **p < 0.01 vs. nonpregnant and normotensive pregnant groups. Proteinuria of all patients from the nonpregnant and normotensive groups was below the detection limit of the measurement

### Decreased α7nAChR was associated with cytokine release from monocytes

**Fig. 1:**
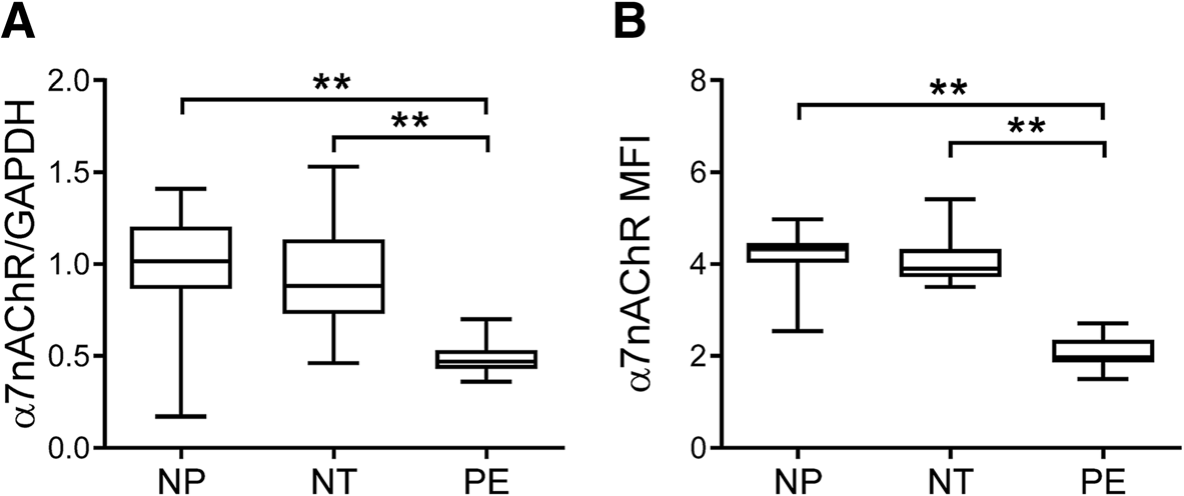
Expression of α7nAChR in peripheral blood monocytes. The mRNA (**a**) and surface protein (**b**) expression of α7nAChR in peripheral blood monocytes isolated from nonpregnant (NP), normotensive pregnant (NT), and preeclamptic (PE) women was measured by RT-PCR and flow cytometry, respectively. MFI: mean fluorescence intensity. **p < 0.01 (one-way ANOVA followed by Bonferroni post-test)

Both surface protein expression and mRNA expression levels of α7nAChR in peripheral blood monocytes from preeclamptic women were significantly lower than those of nonpregnant and normotensive pregnant women (both *p* < 0.01), while levels of α7nAChR in monocytes had no difference between nonpregnant and normotensive pregnant women (Fig. 1A and B). Levels of α7nAChR in monocytes from severe preeclamptic women trended to be, but not significantly, lower than that from mild preeclamptic women (1.95 ± 0.20 vs. 2.14 ± 0.36, *p* = 0.08, student *t*-test).

**Fig. 2:**
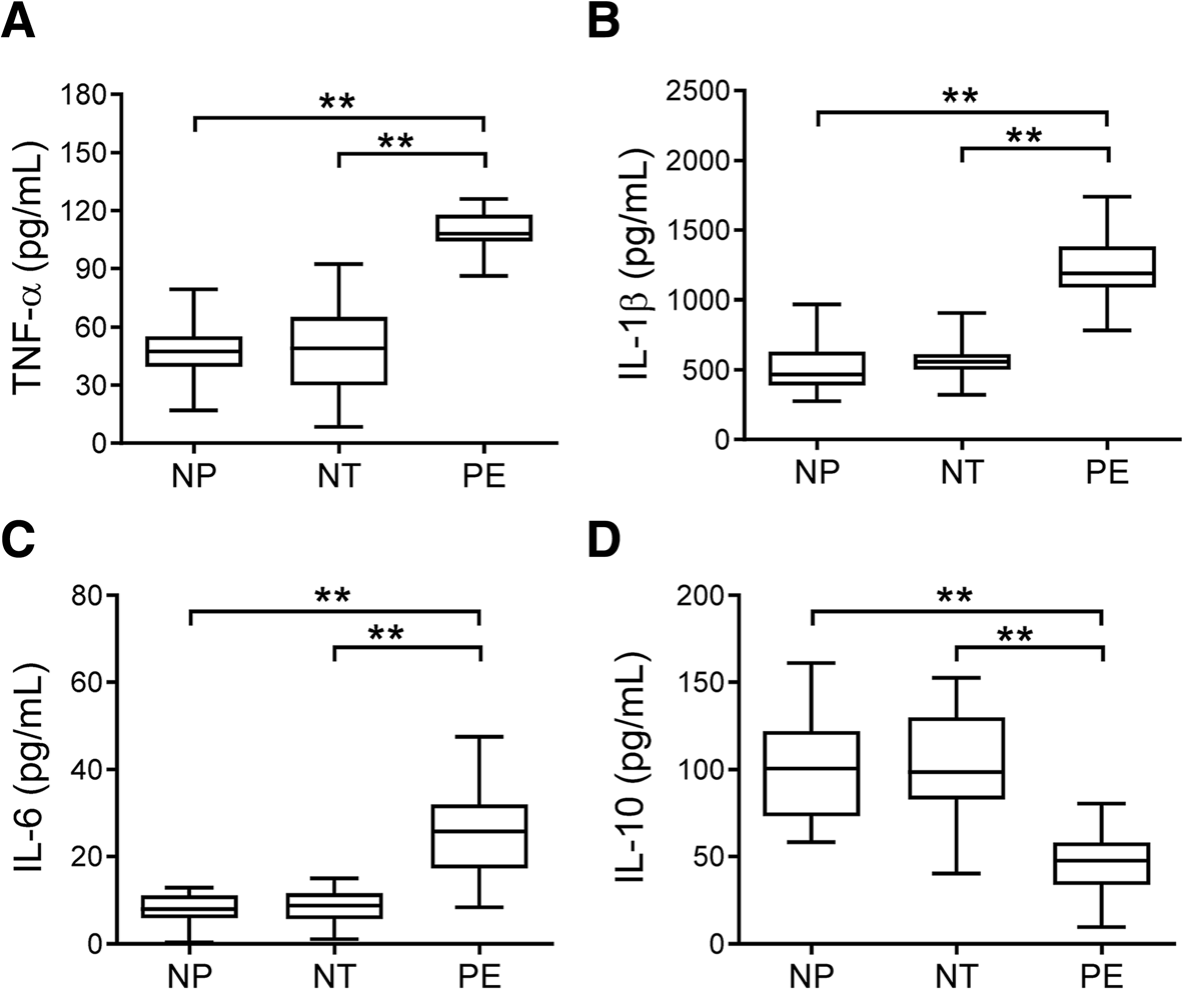
Levels of cytokines in culture medium of isolated monocytes. The protein levels of TNF-α (**a**), IL-1β (**b**), IL-6 (**c**), and IL-10 (D) in culture medium of monocytes isolated from the peripheral blood of nonpregnant (NP), normotensive pregnant (NT), and preeclamptic (PE) women were measured. **p < 0.01 (one-way ANOVA followed by Bonferroni post-test)

**Fig. 3:**
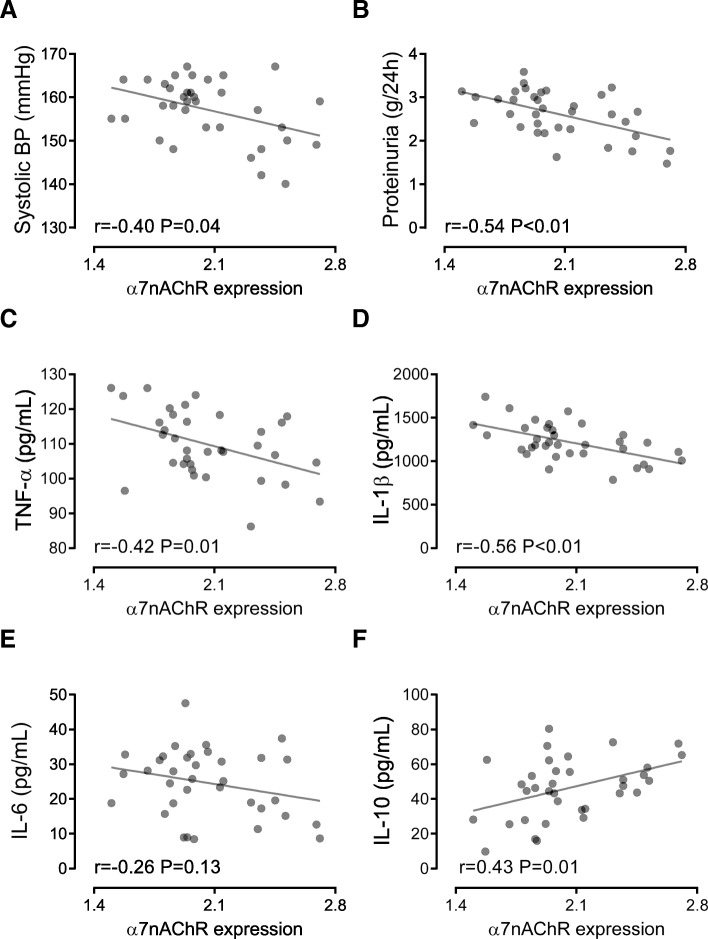
Association between α7nAChR and disease severity and cytokine levels in preeclampsia. The associations of the surface α7nAChR protein expression levels with systolic blood pressure (BP) (**a**) and proteinuria (**b**) of the preeclamptic women, and TNF-α (**c**), IL-1β (**d**), IL-6 (**e**), and IL-10 (**f**) levels in culture medium of monocytes isolated from the preeclamptic women were calculated. Pearson correlation and linear regression were performed

Levels of TNF-α, IL-1β, and IL-6 were increased but IL-10 was decreased in culture medium of monocytes from women with preeclampsia compared with those from nonpregnant and normotensive pregnant women (all *p* < 0.01, Fig. 2A-D). There was no difference in the levels of TNF-α, IL-1β, IL-6, and IL-10 between nonpregnant and normotensive pregnant women (Fig. 2A-D). Importantly, the protein expression levels of α7nAChR in monocytes were negatively correlated with levels of systolic blood pressure (r = − 0.40, *p* = 0.04, Fig. 3A), proteinuria (r = − 0.54, *p* < 0.01, Fig. 3B), TNF-α (r = − 0.42, *p* = 0.01, Fig. 3C), and IL-1β (r = − 0.56, *p* < 0.01, Fig. 3D), while positively correlated with the levels of IL-10 (r = 0.43, *p* = 0.01, Fig. 3F). By contrast, there was no statistically significant correlation between the α7nAChR and IL-6 protein levels (r = − 0.26, *p* = 0.13, Fig. 3E).

### Stimulated cytokine release in monocytes

**Fig. 4:**
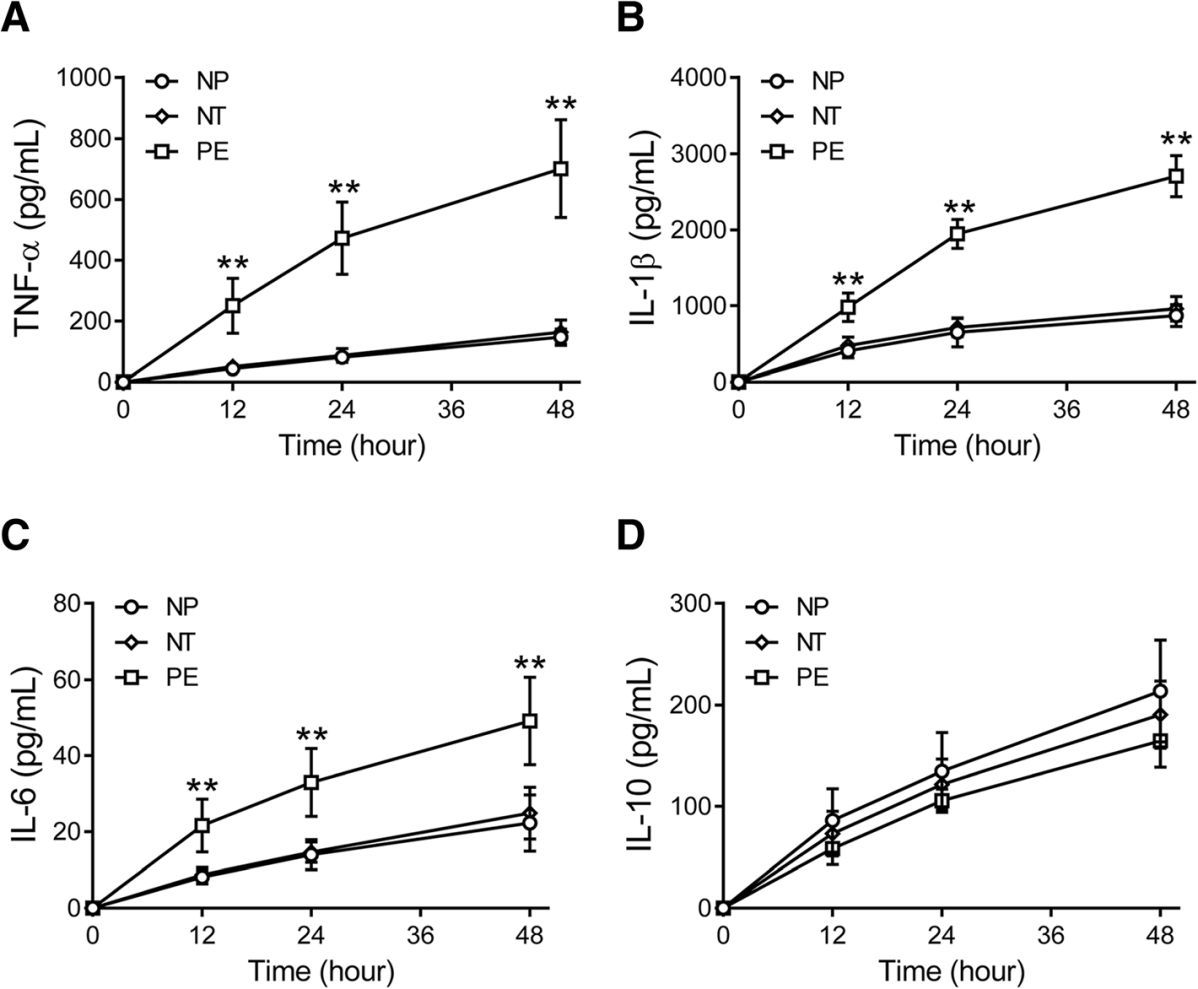
Lipopolysaccharides (LPS)-induced cytokine release from isolated monocytes. Peripheral blood monocytes were isolated from nonpregnant (NP), normotensive pregnant (NT), and preeclamptic (PE) women and stimulated with LPS for 12, 24, or 48 h, then TNF-α (**a**), IL-1β (**b**), IL-6 (**c**), and IL-10 (**d**) levels in the supernatant were measured. **p < 0.01 vs. NP or NT groups at the same time points (one-way ANOVA followed by Bonferroni post-test)

TNF-α, IL-1β, IL-6, and IL-10 were induced by LPS in peripheral blood monocytes isolated from nonpregnant, normotensive pregnant, and preeclamptic women (Fig. 4A-D). At 12, 24, and 48 h after stimulation with LPS, the levels of TNF-α (Fig. 4A), IL-1β (Fig. 4B), and IL-6 (Fig. 4C) in culture medium of monocytes from preeclamptic women were significantly higher than those from nonpregnant and normotensive pregnant women (all *p* < 0.01). However, LPS-induced IL-10 expression levels in culture medium of monocytes were not different among the three groups (Fig. 4D).

### Activation of α7nAChR partially normalized cytokine expression

**Fig. 5:**
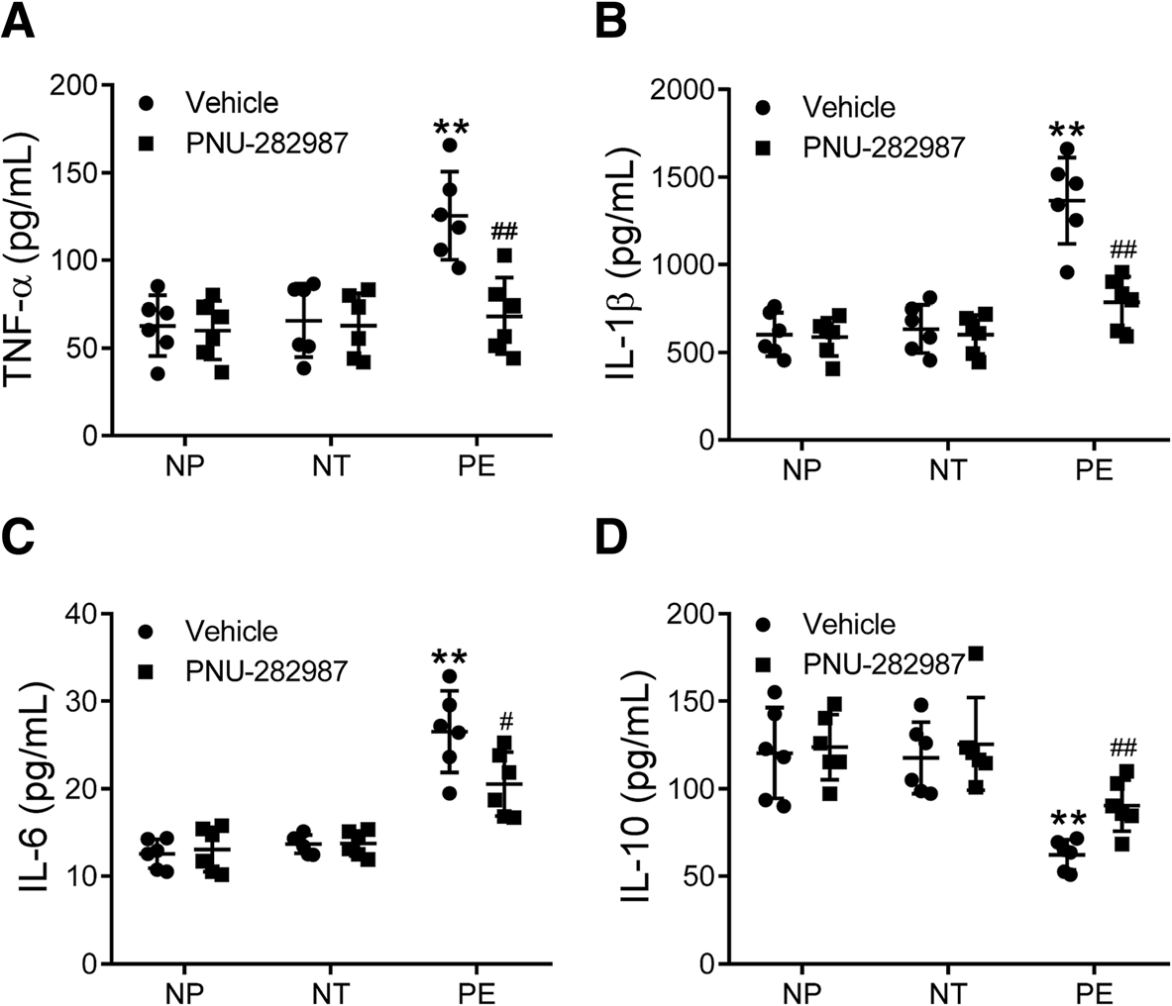
Effects of α7nAChR activation on monocyte cytokine release. Peripheral blood monocytes isolated from nonpregnant (NP), normotensive pregnant (NT), and preeclamptic (PE) women were treated with or without an α7nAChR agonist PNU-282987 for 24 h, then TNF-α (**a**), IL-1β (**b**), IL-6 (**c**), and IL-10 (**d**) levels in the supernatant were measured. **p < 0.01 vs. the vehicle group of the NP and NT groups; ^#^*p* < 0.05, ^##^*p* < 0.01 vs. the PE group treated with vehicle (two-way ANOVA followed by Bonferroni post-test, groups of NP, NT and PE were entered as the between-subjects factor, treatments of vehicle and PNU-282987 were entered as the within-subjects factor)

Monocytes from the three groups of women were treated with the α7nAChR agonist PNU-282987. The increase of TNF-α (Fig. 5A), IL-1β (Fig. 5B), and IL-6 (Fig. 5C) levels in culture medium of monocytes from preeclamptic women was significantly attenuated by PNU-282987 treatment (all *p* < 0.01). Furthermore, the decrease of IL-10 levels in preeclamptic women was also partially reversed by PNU-282987 (Fig. 5D, *p* < 0.01).

### NF-κB transcription activity

**Fig. 6:**
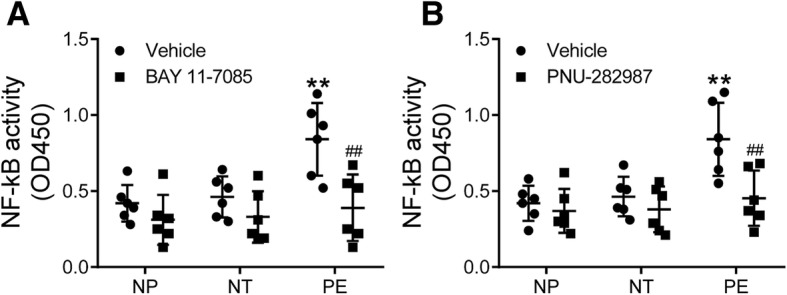
NF-κB activity. Peripheral blood monocytes isolated from nonpregnant (NP), normotensive pregnant (NT), and preeclamptic (PE) women were treated with or without an NF-κB inhibitor BAY 11–7085 (**a**) or α7nAChR agonist PNU-282987 (**b**) for 24 h, then the DNA binding activity of NF-κB was measured. **p < 0.01 vs. the vehicle group of the NP and NT groups; ^##^*p* < 0.01 vs. the PE group treated with vehicle (two-way ANOVA followed by Bonferroni post-test, groups of NP, NT and PE were entered as the between-subjects factor, treatments of vehicle and BAY 11–7085 or PNU-282987 were entered as the within-subjects factor)

The DNA binding activity of NF-κB was enhanced in monocytes from preeclamptic women compared with those from nonpregnant and normotensive pregnant women (*p* < 0.01), and this enhanced activity was abolished by BAY 11–7085 treatment (*p* < 0.01) (Fig. 6A). Interestingly, the enhanced NF-κB transcription activity in monocytes from preeclamptic women was also reversed by PNU-282987 treatment (*p* < 0.01, Fig. 6B).

## Discussion

In this study, we demonstrated a significant association between preeclampsia and downregulation of α7nAChR in peripheral blood monocytes, suggesting that preeclampsia is related to impaired cholinergic anti-inflammatory pathway. Furthermore, we found that the α7nAChR expression levels were closely correlated with the severity of preeclampsia in terms of blood pressure and proteinuria levels. Previously, α7nAChR downregulation was identified in inflammation-related diseases, especially in other types of hypertension [12]. The present study for the first time linked α7nAChR downregulation in circulatory monocytes to pregnancy-induced hypertension. This result is different from previous studies which demonstrated that α7nAChR was upregulated in the placental tissue of women with preeclampsia [21–23]. This difference suggests that the change of α7nAChR expression in preeclampsia is tissue specific.

It is also possible that the downregulation of α7nAChR in circulatory monocytes we found in the present study may not reflect the overall state of the α7nAChR expression in pregnant women with preeclampsia. Anyway, these findings suggest that downregulation of α7nAChR may be associated with the development of pregnancy-induced hypertension and preeclampsia. It is still unclear whether downregulation of α7nAChR precedes onset of preeclampsia. Further studies addressing this question may help to reveal a possible causative relationship between α7nAChR and preeclampsia. It was reported that knockout of α7nAChR exacerbated hypertension in mouse 2-kidney 1-clip model and worsened hypertensive renal injury [28], suggesting that impairment of the cholinergic anti-inflammatory pathway predisposes to hypertension and target organ damage. However, whether α7nAChR downregulation is required for preeclampsia needs further investigation.

α7nAChR may serve as a therapeutic target for pregnancy-induced hypertension and preeclampsia. Animal studies demonstrated that activation of α7nAChR with nicotine significantly attenuated preeclampsia-like symptoms induced by LPS in pregnant rats [20]. α7nAChR agonists also demonstrated anti-hypertensive and kidney-protective effects in other animal models. For example, activation of α7nAChR with nicotine lowered blood pressure in a mouse model of lupus-induced hypertension [10, 29]. However, chronic administration with the α7nAChR agonist PNU-282987 did not significantly alter arterial blood pressure of spontaneously hypertensive rats [28]. In contrast, removal of the cholinergic-sympathetic pathway by coeliac vagotomy prevented angiotensin II-induced hypertension in mice [30]. Therefore, the blood pressure-regulatory role of α7nAChR is various and model-dependent. The therapeutic effects of α7nAChR on pregnancy-induced hypertension and preeclampsia are still elusive.

In the present study, we demonstrated that α7nAChR expression levels were associated with levels of pro-inflammatory cytokines (TNF-α and IL-1β) as well as the anti-inflammatory cytokine (IL-10). Previous studies showed that activation of α7nAChR regulated polarization of macrophages and promoted conversion of classically activated, pro-inflammatory M1 to alternatively activated, anti-inflammatory M2 macrophages [31–33]. Taken together, these findings suggest that downregulation of α7nAChR may polarize more M1 but less M2 macrophages, resulting in increased M1 cytokines (TNF-α and IL-1β) and decreased M2 cytokines such as IL-10. In this study, the α7nAChR expression level in monocytes was not associated with the IL-6 level. As reported, IL-6 acts as both a pro-inflammatory and an anti-inflammatory cytokine and can be synthesized and released by both M1 and M2 macrophages [34–36]. This may be a reason for the nonsignificant association between α7nAChR and IL-6 in monocytes.

Not only the baseline release of pro-inflammatory cytokines from peripheral blood monocytes was increased in preeclampsia, LPS-induced TNF-α, IL-1β, and IL-6 release was also enhanced in women with preeclampsia. LPS-induced cytokine synthesis and release are majorly mediated by activation of NF-κB [37, 38]. Therefore, our findings suggest that the NF-κB pathway is primed to synthesize pro-inflammatory cytokines in monocytes in preeclampsia. This speculation was confirmed by NF-κB activity measurements. LPS stimulation may be different from the inflammatory state in preeclampsia as IL-10 was upregulated by LPS while was downregulated in preeclampsia, which might be why LPS-induced IL-10 was not enhanced in preeclampsia.

Our in vitro experiments demonstrated that activation of α7nAChR with PNU-282987 inhibited NF-κB activation and corrected pro- and anti-inflammatory cytokine expression in monocytes from preeclamptic women. These findings suggest that PNU-282987-caused reverse of cytokine expression is most likely mediated by suppressing the NF-κB pathway.

One of the limitations of the present study is that we could not provide a causative relationship between α7nAChR downregulation and preeclampsia. A future longitudinal study with multiple test timepoints may help to establish the role of α7nAChR downregulation in the development of preeclampsia. We failed to show a significant downregulation of α7nAChR in severe preeclampsia compared with mild preeclampsia, which might be due to the relatively small sample size.

## Conclusion

In conclusion, the findings in this study indicate that downregulation of α7nAChR in peripheral blood monocytes may be associated with the development of pregnancy-induced hypertension and preeclampsia through activating NF-κB and increasing pro-inflammatory and decreasing anti-inflammatory cytokine release.

## Abbreviations

IL: Interleukin
LPS: Lipopolysaccharides
NF-κB: Nuclear factor-κB
NP: Nonpregnant
NT: Normotensive pregnant
PE: Preeclamptic
PNU: PNU-282987
TNF-α: Tumor necrosis factor-alpha
α7nAChR: α7 nicotinic acetylcholine receptor
